# Advances in Hypertension Management: Insights from the Latest European Guidelines

**DOI:** 10.3390/jcdd12040155

**Published:** 2025-04-14

**Authors:** Marco Zuin, Chiara Tognola, Alessandro Maloberti, Gianfranco Parati, Stefania Angela Di Fusco, Vered Gil Ad, Donatella Armata, Chiara Dalla Valle, Furio Colivicchi, Claudio Bilato, Massimo Grimaldi, Fabrizio Oliva, Pier Luigi Temporelli

**Affiliations:** 1 Department of Translational Medicine, University of Ferrara, 44121 Ferrara, Italy; 2 Department of Cardio-Thoraco-Vascular Sciences and Public Health, University of Padova, 35128 Padua, Italy; 3 Department of Cardiology, Madre Teresa di Calcutta Hospital, AULSS 6, Ospedali Riuniti Padova Sud, 35043 Monselice, Italy; 4 Unit of Cardiology 4, ASST GOM Niguarda, 20162 Milano, Italy; chiara.tognola@ospedaleniguarda.it (C.T.); alessandro.maloberti@unimib.it (A.M.); 5 Department of Medicine and Surgery, Università degli studi Milano-Bicocca, 20133 Milano, Italy; gianfranco.parati@unimib.it; 6 Istituto di Ricovero e Cura a Carattere Scientifico (IRCCS), Istituto Auxologico Italiano, 20149 Milan, Italy; 7 U.O.C. Cardiologia Clinica e Riabilitativa, Presidio Ospedaliero San Filippo Neri—ASL Roma 1, 00161 Roma, Italyfurio.colivicchi@aslroma1.it (F.C.); 8 Unit of Cardiology, Ospedale Policlinico San Martino, 16132 Genova, Italy; veredgilad@gmail.com; 9 Fondazione G. Giglio, 90015 Cefalù, Italy; donatella.armata@hsrgiglio.it; 10 Department of Cardiology, West Vicenza General Hospital, 36071 Arzignano, Italy; chiara.dallavalle@aulss8.veneto.it (C.D.V.); claudio.bilato@aulss8.veneto.it (C.B.); 11 U.O.C. Cardiologia-UTIC, Ospedale Miulli, Acquaviva delle Fonti, 70021 Bari, Italy; m.grimaldi@miulli.it; 12 Unità di Cure Intensive Cardiologiche, Cardiologia 1-Emodinamica, Dipartimento Cardiotoracovascolare “A. De Gasperis”, ASST Grande Ospedale Metropolitano Niguarda, 20133 Milano, Italy; fabrizio.oliva@ospedaleniguarda.it; 13 Associazione Nazionale Medici Cardiologi Ospedalieri (ANMCO), 50121 Firenze, Italy; 14 Divisione di Cardiologia Riabilitativa, Istituti Clinici Scientifici Maugeri, IRCCS, 28013 Gattico-Veruno, Italy; pierluigi.temporelli@icsmaugeri.it

**Keywords:** arterial hypertension, diagnosis, treatment, guidelines

## Abstract

Arterial hypertension is one of the most common and preventable risk factors for cardiovascular disease and its related mortality. Currently, the prevalence of hypertension in different European countries appears to be around 30–45% of the general population, with a steep increase with ageing. Recent European guidelines have introduced novel recommendations for the management and treatment of hypertensive patients, with direct implications in daily clinical practice. Therefore, in this focused review, we will provide answers to the most common questions regarding the diagnosis, management and treatment of arterial hypertension according to the latest available European guidelines.

## 1. Introduction

Arterial hypertension (HT) represents one of the most common and preventable risk factors for cardiovascular diseases (CVDs) and related mortality [1]. Recent European epidemiological data estimated a prevalence of HT of around 30–45% in the general population, which increases with age [2]. Despite available therapeutic options, fewer than half of hypertensive patients achieve the recommended blood pressure (BP) levels for their cardiovascular risk category [3,4], resulting in significant hospitalization, morbidity, mortality, and cost implications for the National Health Care Systems. Over the past two years, both the European Society of Hypertension (ESH) [5] and the European Society of Cardiology (ESC) [6] have updated treatment guidelines for hypertensive patients, highlighting both similarities and differences. However, these new management strategies can create uncertainty in daily clinical practice. Therefore, this review aims to address common questions regarding the diagnosis, management, and treatment of arterial hypertension based on the latest European guidelines on the management of HT.

## 2. ESH and ESC Guidelines: A Comparative Analysis

**Figure 1:**
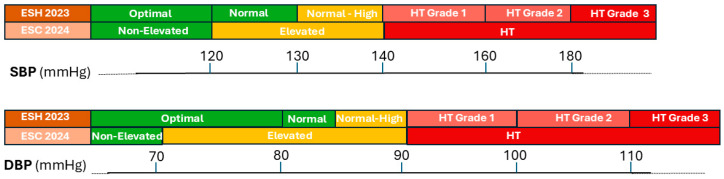
Differences in the classification of arterial hypertension between the Guidelines of the European Society of Hypertension (ESH) and the European Society of Cardiology (ESC). SBP: Systolic Blood Pressure; DBP: Diastolic Blood Pressure; HT: Hypertension.

The 2023 European Society of Hypertension (ESH) guidelines and the 2024 European Society of Cardiology (ESC) guidelines exhibit significant differences in classification, criteria for initiating therapy, and recommended therapeutic approaches [5,6]. Specifically, the ESC guidelines introduce the term “elevated blood pressure” for patients with systolic BP (SBP) between 120 and 139 mmHg and diastolic BP (DBP) between 80 and 89 mmHg [6]. This category corresponds to the ESH-defined categories of “normal” (120–129/80–84 mmHg) and “high-normal” (130–139/85–89 mmHg) [5]. Conversely, both guidelines maintain a threshold of 140/90 mmHg for diagnosing arterial hypertension, but the ESC guidelines [6] no longer adopt sub-classification based on the degree of hypertension. Below 120/80 mmHg, both guidelines agree on normality, although the ESC prefers the term “non-elevated” over the optimal term used by ESH, acknowledging that cardiovascular events can occur at these levels [5,6] (Figure 1). For patients with high-normal blood pressure values, the ESH guidelines [5] recommend therapy only in cases of very high cardiovascular risk or previous myocardial infarction. On the other hand, the ESC guidelines recommend therapy in patients at high cardiovascular risk defined by known cardiovascular disease, hypertensive organ damage, SCORE-2 ≥ 10%, or other specific factors (e.g., HIV infection, rheumatological diseases) [6]. In the absence of these conditions, they suggest a thorough evaluation of non-conventional factors related to sex and ethnicity, coronary calcium score, cardiac biomarkers, or carotid–femoral plaque detection to further improve cardiovascular risk classification [6]. The primary difference in therapeutic approach is that both guidelines recommend initial dual-drug combination therapy; however, while the ESH guidelines [5] suggest transitioning to triple combination therapy only after titrating two molecules in a single pill to the maximum dosage, the ESC guidelines [6] recommend a rapid transition to low-dose triple combination therapy if blood pressure targets are not achieved. Emerging technologies like cuffless blood pressure monitors enable continuous BP tracking, providing valuable insights into nocturnal patterns and “time in target range”. However, the ESC and ESH guidelines do not yet include formal recommendations for these devices, underscoring the need for further validation studies and standardization. While telehealth platforms and mobile applications have shown promise in enhancing patient engagement, real-world evidence of their long-term clinical impact remains limited. Looking ahead, future guidelines may benefit from integrating digital biomarkers to support more personalized and data-driven hypertension management.

## 3. European and American Guidelines: What Are the Differences?

In 2017, the American guidelines redefined hypertension as ≥130/80 mmHg, aiming to identify high-risk individuals earlier [7]. In contrast, ESC and ESH guidelines retained the traditional threshold of ≥140/90 mmHg, citing concerns about overmedicalization and a lack of robust evidence supporting lower thresholds in low-risk populations [5,6]. As a result, nearly 30% more adults would be classified as hypertensive under the American College of Cardiology/American Heart Association (ACC/AHA) criteria compared to ESC and ESH definitions [5,6]. Despite this difference in diagnostic cutoffs, both guidelines recommend initiating pharmacological treatment at ≥130/80 mmHg in patients with established cardiovascular disease (CVD) or elevated cardiovascular risk. However, risk stratification methods differ: the ACC/AHA defines high risk as a ≥10% 10-year atherosclerotic cardiovascular disease (ASCVD) risk [7], while the ESC and ESH employ the SCORE-2 system, which may identify fewer patients as high-risk [5,6]. In terms of treatment, both guidelines recommend angiotensin-converting enzyme inhibitors (ACE-I), angiotensin receptor blockers (ARBs), thiazide diuretics, and CCBs as first-line agents. However, the ESC and ESH guidelines permit beta-blockers as initial therapy in certain conditions, such as atrial fibrillation or chronic obstructive pulmonary disease (COPD), whereas the ACC/AHA restricts their use to heart failure or ischemic heart disease [7]. The ACC/AHA also recommends initial dual therapy for patients with blood pressure >20/10 mmHg above target or for Black individuals, while the ESC/ESH favors starting most patients on single-pill combination therapy to enhance adherence. Notably, the ESC and ESH guidelines include device-based therapies such as renal denervation for resistant hypertension (RH) (Class II recommendation), a strategy not currently addressed by the ACC/AHA [5,6,7]. When managing older adults, the ESC and ESH recommend more conservative systolic targets (e.g., 140–150 mmHg for patients aged ≥80 years) to minimize adverse outcomes like hypotension. In contrast, the ACC/AHA maintains a uniform target of <130/80 mmHg regardless of age [7]. Both guidelines emphasize accurate blood pressure measurement, lifestyle modifications, and individualized, risk-based treatment strategies. Adherence to guideline-recommended targets is associated with significantly improved blood pressure control rates—up to 23% higher in clinical practice. For instance, strict adherence to ESC and ESH guidelines in older adults helps prevent overtreatment, while the ACC/AHA’s lower thresholds may offer greater cardiovascular protection in younger, high-risk populations [5,6,7]. Going forward, harmonizing these international guidelines may help balance the benefits and risks of treatment thresholds, incorporating emerging evidence on personalized care and novel therapeutic options.

## 4. Approaching the Newly Diagnosed Hypertensive Patient

**Figure 2:**
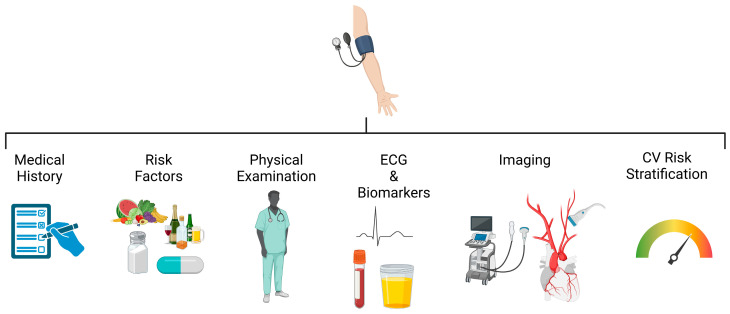
Algorithm for the outpatient evaluation of a patient with newly diagnosed arterial hypertension.

Hypertensive patients exhibit significant heterogeneity in their phenotypic presentation, risk factors, comorbidities, and the presence and severity of organ damage. Consequently, the approach to newly diagnosed hypertensive patients should be based on standardized criteria that integrate with a holistic view of the patient (Figure 2). The initial clinical evaluation must include a thorough medical history aimed at investigating not only the patient’s clinical background but also physiological aspects such as dietary habits, salt intake, alcohol or liquorice consumption, smoking status, and the use of medications that may influence blood pressure. Then, a comprehensive physical examination must be performed to identify suggestive elements of secondary hypertension and any overt organ damage. Cardiovascular risk stratification must be integrated with the evaluation of biochemical tests, including plasma creatinine and 24 h creatinine clearance, microalbuminuria, electrolytes, and a complete lipid profile. If a secondary HT is suspected, it is necessary to investigate thyroid function, plasma and urinary cortisol levels, plasma renin activity and aldosterone, and 24 h urinary catecholamines and metanephrines. A 12-lead electrocardiogram (ECG) is always recommended during clinical evaluation. Moreover, it should also be recommended to perform a transthoracic echocardiogram, especially during the first clinical evaluation, to identify any hypertensive organ damage. The measurement of BP during the visit represents a crucial phase: according to guidelines, it should be performed in triplicate on the dominant arm. All these evaluations allow for precise cardiovascular risk stratification using SCORE-2, which is fundamental for subsequent therapeutic decisions [6].

## 5. Hypertension-Mediated Organ Damage

**Figure 3:**
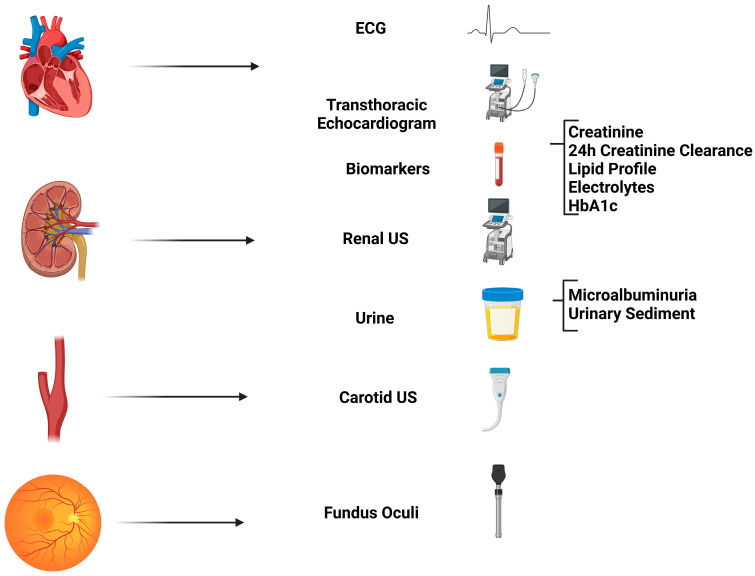
Evaluation of organ damage mediated by arterial hypertension. TSA: Supra-aortic Trunks. US: Ultrasound.

Hypertension-mediated organ damage (HMOD) refers to structural and functional alterations affecting major arteries and target organs, including the heart, kidneys, central nervous system, and retina. This damage is secondary to elevated blood pressure and is frequently observed in cases of severe or long-standing HT. However, due to advancements in imaging techniques and increased clinical vigilance, HMOD is increasingly detected even in less severe HT [8]. Identifying HMOD is crucial for cardiovascular risk stratification, as its presence significantly increases the incidence of major adverse events such as myocardial infarction, stroke, renal failure, and cardiovascular mortality [9]. Left ventricular hypertrophy (LVH) represents the physiological response to chronic pressure overload. Transthoracic echocardiography is the primary tool for quantifying left ventricular mass and assessing other hypertension-related cardiac damage, including diastolic function, ascending aortic dilatation, or left atrial enlargement [10]. Vascular damage can be evaluated through arterial stiffness by measuring carotid–femoral pulse wave velocity; a value exceeding 10 m/s indicates significantly increased arterial stiffness [11]. Additionally, the presence of atheromatous plaques in the supra-aortic trunks or an ankle-brachial index <0.9 are indicative of atherosclerosis, which increases cardiovascular risk [12]. Renal damage initially manifests as microalbuminuria (>30 mg/24 h), while a glomerular filtration rate below 60 mL/min indicates advanced renal involvement. Finally, central nervous system involvement can be assessed using CT or MRI scans in specific cases (Figure 3). The 2024 ESC guidelines stress the importance of a thorough evaluation of HMOD through standardized assessments. These include an ECG to detect left ventricular hypertrophy (LVH), an echocardiography for structural heart evaluation, urine albumin-to-creatinine ratio to identify kidney damage, and a fundoscopy for signs of hypertensive retinopathy [6]. Management focuses on aggressive blood pressure control, with an initial systolic target of 120–129 mmHg for most patients, alongside individualized therapy to prevent or reverse HMOD progression. First-line treatment includes renin–angiotensin–aldosterone system (RAAS) inhibitors for patients with albuminuria or LVH, combined with lifestyle modifications such as sodium restriction and weight loss. The guidelines also recommend ambulatory blood pressure monitoring to confirm the diagnosis and assess treatment efficacy, particularly for cases of suspected white-coat or masked hypertension, to ensure accurate risk stratification and avoid under- or over-treatment. These updated guidelines refine those from the ESH by incorporating out-of-office BP measurements and personalized risk algorithms while maintaining core recommendations for routine HMOD screening in all hypertensive patients [5].

## 6. The Role of 24 h Ambulatory Monitoring and Home Blood Pressure Monitoring

Automated oscillometric BP measurements can be utilized outside clinical settings through 24 h ambulatory blood pressure monitoring (ABPM; Table 1) and home blood pressure monitoring (HBPM; Table 2) [13,14]. These methods significantly enhance hypertension management by identifying white-coat hypertension and masked hypertension (see related section). ABPM provides comprehensive 24 h data, including circadian profiles and nocturnal dipping patterns, enabling clinicians to verify 24 h BP control in treated patients. Similarly, HBPM reduces variability from isolated measurements by assessing BP across multiple days, offering insights into long-term trends [15]. Out-of-office BP measurements enable personalized pharmacological treatment adjustments based on real-world BP patterns, addressing both therapeutic inefficacy and excessive reductions. HBPM actively involves patients, fostering awareness and improving treatment adherence. Both HBPM and ABPM assess BP variability, which is independently associated with elevated cardiovascular risk [16]. Cuff-dependent devices provide static, snapshot measurements under controlled conditions, failing to capture dynamic BP fluctuations during daily activities. Common technical limitations include improper cuff sizing, shape, or placement errors, as well as discomfort during cuff inflation, particularly during sleep or work. Wearable cuffless devices estimate BP using sensors that detect biological signals (e.g., photoplethysmography) calibrated against conventional measurements. However, key challenges persist: the accuracy of these devices remains unproven due to inadequate validation protocols, and there is a lack of standardization regarding performance benchmarks. Most require periodic recalibration using standard BP measurements, and some rely on user-specific data (age, sex). Cuffless devices track BP variations relative to calibration baselines rather than measuring absolute values. Thus, they are not recommended for hypertension diagnosis or clinical management until further validation. Incorporating out-of-office BP monitoring into practice enhances diagnostic accuracy and therapeutic precision. While current diagnostic thresholds and treatment targets remain anchored to conventional measurements [6,17], recent guidelines emphasize the prognostic value of out-of-office BP. Validated automated devices, such as those listed on STRIDE BP (www.stridebp.org), accessed 6 March 2025) an initiative endorsed by the European Society of Hypertension (ESH), the International Society of Hypertension (ISH), and the World Hypertension League (WHL), should be prioritized to ensure reliability [18].

## 7. Recommended Therapies and Emerging Strategies

**Figure 4:**
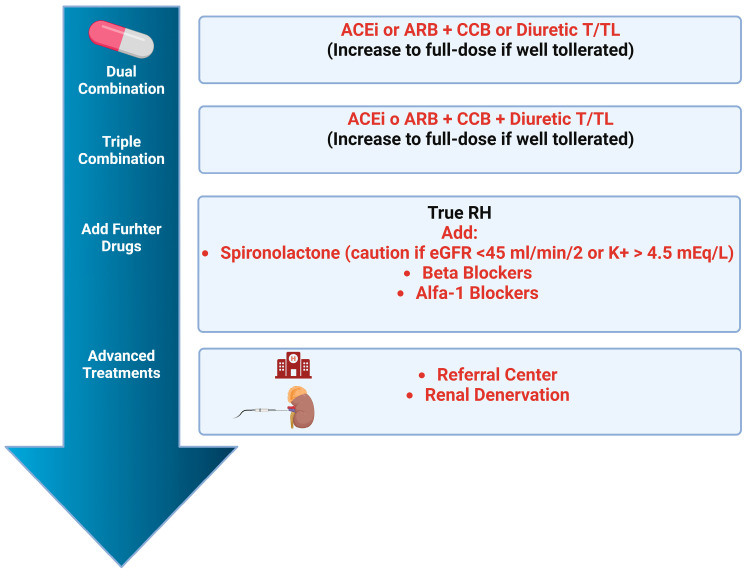
Therapeutic algorithm for antihypertensive therapy. ACEi: ACE Inhibitors; CCB: Calcium Channel Blockers; Diuretics T: Thiazide Diuretics; Diuretics TL: Thiazide-like Diuretics.

Both the 2023 ESH and the 2024 ESC guidelines recommend a combined approach for managing arterial hypertension, incorporating lifestyle modifications and pharmacological therapy [5,6]. From a pharmacological perspective, the primary classes of drugs with strong evidence for reducing cardiovascular events include ACE-I and angiotensin II receptor blockers (ARB), dihydropyridine calcium channel blockers (CCB), and diuretics (notably thiazide and thiazide-like diuretics such as hydrochlorothiazide, chlorthalidone, and indapamide). These medications are recommended as first-line treatments, typically starting with a combination of two molecules (e.g., ACE-I or ARB plus CCB or diuretics), with a progressive increase to a triple combination if necessary. Maintaining single-pill combinations is encouraged to enhance patient adherence [6]. Additional pharmacological options, often used in resistant hypertension or when intolerance to first-line treatments occurs, include aldosterone antagonists, alpha-blockers, and centrally acting agents. While the ESC guidelines suggest using beta-blockers [19] preferentially in specific contexts such as angina pectoris, chronic heart failure, or post-myocardial infarction, the ESH guidelines propose that beta-blockers can be added at any step of therapy [5]. Beta-blockers exhibit significant differences within the class; for example, bisoprolol, atenolol, and metoprolol can be used for both blood pressure control and other indications like heart rate control or arrhythmia management. Nebivolol is noted for its pronounced antihypertensive efficacy due to its ability to stimulate nitric oxide release (Figure 4).

## 8. Resistant Hypertension: Definition, Pharmacological and Non-Pharmacological Treatment Strategies

**Figure 5:**
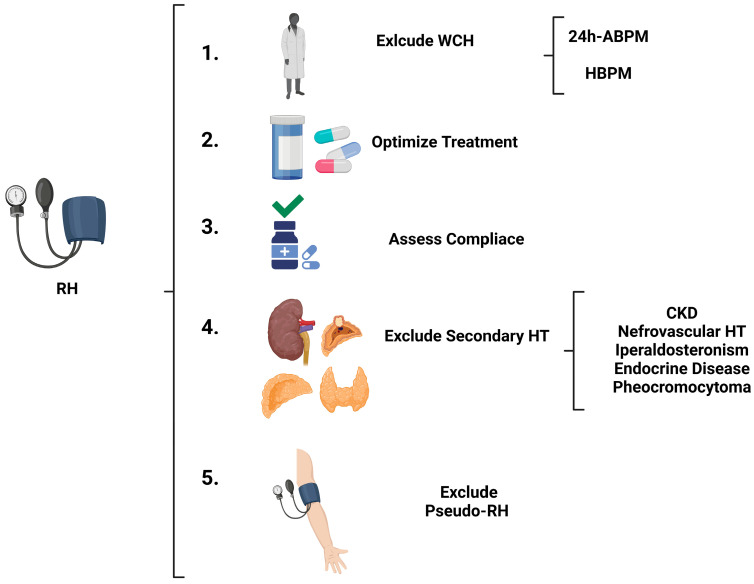
Diagnostic algorithm to exclude resistant arterial hypertension. ABPM: Ambulatory Blood Pressure Monitoring; HBPM: Home Blood Pressure Monitoring; RH: Resistant Hypertension; CKD: Chronic Kidney Disease; HT: Hypertension.

Resistant hypertension (RH) is defined as the persistence of BP values above target levels despite treatment with at least three antihypertensive medications at optimal doses, including a diuretic. It is crucial to differentiate true resistant hypertension from “pseudo-resistance”, often caused by poor adherence to therapy, suboptimal dosing, incorrect blood pressure measurements, or white-coat effect. Historically, the prevalence of resistant hypertension was approximately 21%, but it has decreased to around 5% due to advanced therapeutic combinations and improved diagnosis of pseudo-resistant and secondary forms [20,21]. Both the ESH and ESC guidelines propose similar flow charts for managing patients with RH (Figure 5). In terms of therapeutic strategy, both guidelines suggest the use of aldosterone antagonists, which have proven particularly effective in reducing blood pressure in RT [22]. When aldosterone antagonists are not tolerated or contraindicated (e.g., in patients with an estimated glomerular filtration rate < 30 mL/min) or cause side effects (acute renal failure on chronic, hyperkalemia, gynecomastia), alternatives such as beta-blockers (if not already used for other indications) and other molecules (doxazosin, clonidine, amiloride, methyldopa, hydralazine, loop diuretics) can be considered. Moreover, renal denervation must also be considered for these patients. Guidelines indicate renal denervation for patients with a glomerular filtration rate > 40 mL/min who have resistant hypertension or experience side effects from multiple antihypertensive drugs (difficult-to-treat patients) [5,6]. Recent sham-controlled randomized clinical trials have shown that renal denervation is a safe and effective method for reducing systolic and diastolic blood pressure values in both pharmacologically treated and untreated patients [23,24].

## 9. Lifestyle Modifications for Effective Control of Arterial Hypertension

Lifestyle changes are a fundamental component in the approach to preventing and treating hypertension, as well as improving cardiovascular health, which is why guidelines recommend their implementation as a first-line intervention [6,25]. Among the recommended measures (Table 3) is the reduction of dietary sodium intake. In this regard, it was shown that lowering it below 2.5 g/day is associated with a 20% reduction in cardiovascular events [26]. Additionally, adequate potassium intake is known to reduce blood pressure, with recommendations to consume over 3.5 g/day through fruits and vegetables [25]. The Mediterranean diet and the Dietary Approaches to Stop Hypertension (DASH) diet are dietary regimens proven to be effective in reducing blood pressure and cardiovascular diseases [26,27,28]. The DASH diet emphasizes fruits, vegetables, whole grains, low-fat dairy products, white meat, fish, and vegetable oils, while significantly reducing or eliminating red meat, animal fats, sugar, and alcohol, and limiting salt intake. Although caffeine consumption is not associated with an increased risk of hypertension in the general population, energy drinks with high concentrations of ingredients like taurine and caffeine should be avoided as they increase blood pressure and can lead to acute and chronic cardiovascular complications [29]. In children and adolescents, the consumption of sugary drinks is linked to increased systolic blood pressure values and a higher risk of developing hypertension [30]. Guidelines recommend limiting their intake to no more than 10% of daily energy intake [5,6]. Alcohol consumption, even at low doses (e.g., 10 g/day), increases the risk of hypertension by 14% in men but not in women [31]. Regular aerobic exercise in hypertensive individuals reduces cardiovascular mortality [32]. In obese or overweight subjects, weight loss is associated with reduced blood pressure, with greater benefits from more substantial weight reduction [33]. Weight loss also improves glucose and lipid metabolism, leading to benefits across the cardiovascular and metabolic systems beyond blood pressure reduction [34]. Finally, while chronic tobacco smoking has a relatively marginal effect on blood pressure, the impact of electronic cigarettes appears to be greater [35].

## 10. Hypertension Management in Young and Elderly Patients

Young individuals, previously normotensive, who rapidly develop arterial hypertension or experience uncontrolled blood pressure despite usual therapy should be evaluated for possible secondary hypertension [6]. From an epidemiological perspective, secondary hypertension is more common in young adults, with a prevalence ranging from 15% to 30% [36]. The primary causes of secondary hypertension include drug-induced hypertension and primary aldosteronism. Additionally, the consumption of drugs, dietary supplements, and energy drinks can also be a potential etiology of hypertension. Among medications, estrogen–progestin contraceptives are a leading cause of secondary hypertension in young women and should be avoided in hypertensive young women, with preference given to progestin-only contraceptives. Other forms of secondary hypertension include fibromuscular dysplasia and sleep apnea syndromes, particularly in overweight or obese individuals [5,6]. 

The early diagnosis of HMOD is crucial in individuals under 40 years old with high cardiovascular risk as risk estimation cannot be accurately performed using the SCORE-2 system in this demographic group. An aggressive approach to antihypertensive treatment and risk factor correction is advisable in this group. Both the ESH and ESC guidelines devote particular attention to the evaluation and treatment of elderly patients, defined as those over 80 years old [5,6]. Specifically, the ESH [6] guidelines recommend assessing their functional and autonomy status in daily activities (ADL) using the Katz Index and the Mini-Mental State Examination (MMSE). This approach allows for the identification of three distinct patient groups: autonomous, limited, and fully dependent. Autonomous patients over 80 years old should initiate pharmacological treatment when ambulatory systolic blood pressure (SBP) is ≥160 mmHg, although therapy may also be considered for SBP values between 140 and 159 mmHg. In contrast, non-autonomous and fully dependent patients should only start antihypertensive therapy for SBP ≥ 160 mmHg. The ESC guidelines propose a frailty score based on nine different phenotypes. Both ESC and ESH guidelines recommend initiating antihypertensive therapy with monotherapy in elderly and frail patients, with cautious titration to avoid orthostatic hypotension and potential falls [5]. Therapy should be discontinued for SBP < 120 mmHg in these patients. It is important to note that HMOD in elderly individuals can manifest heterogeneously, involving the central nervous system with symptoms such as vertigo, syncope, worsening visual function, transient ischemic attacks, motor or sensory deficits, cognitive decline, memory loss, or dementia.

## 11. Managing Hypertension in Pregnancy and Puerperium

Monitoring and treating hypertension during pregnancy are crucial to prevent potential maternal and fetal complications. Recent epidemiological data indicate that hypertensive disorders affect approximately 10% of pregnancies worldwide and are the leading cause of maternal, fetal, and neonatal morbidity and mortality [37]. Guidelines identify gestational hypertension, preeclampsia, and chronic hypertension as the primary forms of hypertensive disorders in pregnancy, each with distinct characteristics and specific therapeutic indications. Gestational hypertension is diagnosed when BP is ≥140/90 mmHg on at least two separate occasions after the 20th week of gestation and typically resolves about six weeks postpartum [5,6]. Hypertension diagnosed before the 20th week of gestation is considered chronic, as it pre-exists the pregnancy. Preeclampsia is defined as HT after the 20th week in previously normotensive women, associated with proteinuria (>0.3 g/day), maternal renal, hepatic, neurological, or hematological dysfunction, or uteroplacental dysfunction (fetal growth restriction, abnormal umbilical artery Doppler waveforms, or intrauterine death) [5,6]. Low-dose aspirin (100–150 mg from the 11th to the 35th week) should be administered to pregnant women at a high or moderate risk of developing preeclampsia based on first-trimester combined screening to prevent its onset [5,6]. The definitive treatment for gestational HT and preeclampsia is delivery. Antihypertensive medication in conjunction with lifestyle modifications serves as a bridge until fetal maturity allows for safe delivery. Pharmacological therapy is recommended for all pregnant women with blood pressure ≥ 140/90 mmHg to reduce it below this level. In the first trimester, a physiological decrease in blood pressure is common; thus, in women with chronic hypertension on monotherapy, treatment is often suspended with close subsequent monitoring. For those on combination therapy, medications are replaced with pregnancy-safe alternatives such as extended-release nifedipine, labetalol, and methyldopa upon pregnancy confirmation [5,6]. In cases of preeclampsia, intravenous medications may be necessary, with labetalol being the preferred choice, potentially combined with hydralazine in severe cases. Additionally, magnesium sulfate is indicated as an anticonvulsant for severe preeclampsia and eclampsia. It is essential to avoid teratogenic therapies (e.g., RAS inhibitors and diuretics) in women of childbearing age. If a woman on such treatments desires pregnancy, they should be replaced with non-teratogenic medications at least one month prior. During breastfeeding, nifedipine, labetalol, methyldopa, captopril, enalapril, amlodipine, and metoprolol can be administered. However, there is insufficient safety data regarding the use of other angiotensin II receptor blockers and ACE inhibitors beyond enalapril and captopril [38].

## 12. White-Coat Hypertension and Masked Hypertension

**Figure 6:**
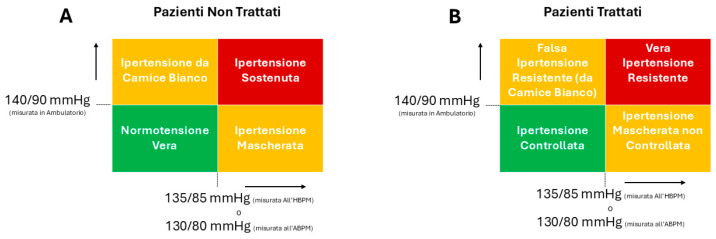
Classification of arterial hypertension in relation to its ambulatory measurement, ambulatory blood pressure monitoring (ABPM), and home blood pressure monitoring (HBPM) in untreated patients (**A**) and treated patients (**B**).

When blood pressure is evaluated using both office and out-of-office measurements, patients can be categorized into four groups (Figure 6, Panel A): normotension (non-elevated blood pressure in both settings), sustained hypertension (elevated blood pressure in both settings), white-coat hypertension (WCH; elevated office blood pressure with normal out-of-office readings), and masked hypertension (MH; normal office blood pressure with elevated out-of-office readings) [39,40,41,42,43]. WCH and MH are relatively common in both untreated and treated hypertensive individuals, with approximately 15–25% of hypertensive patients experiencing WCH and 10–20% experiencing MH, although their reproducibility is limited. In treated patients, different definitions are used, such as uncontrolled white-coat hypertension or masked uncontrolled hypertension (Figure 6, Panel B). These conditions represent unique clinical scenarios requiring approaches distinct from traditional hypertension management. Currently, there are no randomized intervention studies demonstrating treatment benefits, and guideline recommendations are based on expert opinion. Both conditions should always be managed with lifestyle modifications, while pharmacological therapy may be considered in certain situations. For WCH, pharmacological treatment may be warranted if hypertension-mediated organ damage (HMOD) is present, if significant cardiovascular risk factors exist (e.g., diabetes, obesity, family history of early cardiovascular events, nephropathy), or if it progresses to sustain hypertension. MH can be treated pharmacologically if persistent, as it is associated with a cardiovascular risk like that of sustained hypertension, particularly in patients with additional risk factors [5,6].

## 13. Conclusions

In conclusion, the 2024 ESC and the 2023 ESH guidelines both emphasize the importance of managing elevated BP and HT to reduce cardiovascular risk. The 2024 ESC guidelines introduce a new systolic blood pressure target of 120–129 mmHg for most adults, reflecting a shift toward more intensive treatment strategies, albeit with caveats for tolerance and specific patient conditions, such as symptomatic orthostatic hypotension or frailty [5,6]. Both guidelines highlight the value of out-of-office blood pressure measurements and multidisciplinary care approaches. While the ESC guidelines focus on evidence related to fatal and non-fatal cardiovascular disease outcomes, the ESH guidelines provide a comprehensive framework for hypertension management, including lifestyle modifications and pharmacological interventions [5,6]. Overall, these guidelines align in their emphasis on personalized treatment and the integration of novel technologies to enhance blood pressure control but differ in their specific recommendations for blood pressure targets and treatment intensification.

## Figures and Tables

**Table 1:** Clinical indications for ambulatory and home blood pressure monitoring (BP). ABPM: Ambulatory Blood Pressure Monitoring; HBPM: Home Blood Pressure Monitoring. * Using only devices validated according to international protocols.

Indications for 24 h ABPM	Indications for HBPM
Grade I hypertension in office measurements	Long-term monitoring of treated individuals to improve adherence and blood pressure control
Significant increase in office blood pressure without hypertension-mediated organ damage (HMOD)	Patients unwilling or unable to undergo ABPM or those experiencing significant discomfort during recording
Normal-to-high blood pressure in office measurements	Confirmation of white-coat hypertension in untreated or treated individuals *
Normal blood pressure in office measurements in individuals with HMOD or high total cardiovascular risk	Confirmation of masked hypertension in untreated or treated individuals *
Diagnosis of masked hypertension	Pregnancy
Diagnosis of white-coat hypertension	Evaluation of nighttime blood pressure and dipping profile (e.g., in cases of sleep apnea, chronic kidney disease, diabetes, endocrine hypertension, or autonomic dysfunction)
In hypertensive patients under treatment for: Confirmation of uncontrolled and true resistant hypertension Evaluation of 24 h blood pressure control (especially in high-risk patients) Assessment of symptoms suggestive of hypotension (especially in elderly patients) Exaggerated blood pressure response to exercise	

**Table 2:** Recommendations for home blood pressure monitoring (HBPM).

Pre-Measurement Conditions	Quiet Room: Ensure the room is quiet and has a comfortable temperature.
	Avoid Stimulants: No smoking, caffeine, food, or exercise in the 30 min preceding measurement.
	Rest: Remain seated and relaxed for 3–5 min.
	Silence: Do not speak during or between measurements.
Position During Measurement	Seated: Sit with back supported by a chair.
	Leg Position: Legs should not be crossed, with feet flat on the floor.
	Arm Position: Bare arm should be supported on a table, with the middle of the arm at heart level.
Cuff Instructions	Cuff Size: Select the appropriate cuff size based on arm circumference according to device instructions.
	Cuff Placement: Wrap the cuff around the bare arm following device instructions (usually on the left arm).
Patient Instructions	Device Selection: Use a reliable and validated device.
	Measurement Conditions: Ensure correct conditions and posture for measurement.
	Measurement Schedule: Plan measurements before office visits and between visits.
	Interpretation: Inform patients about normal blood pressure variability.
	Action Plan: Advise on actions to take if blood pressure is too high or too low.
Recommendations Before a Scheduled Visit	Duration: Measure for 7 days (at least 3 days).
	Timing: Measure in the morning and evening.
	Pre-Medication and Meals: Measure before taking medication (if treated) and before meals.
	Measurement Protocol: Take two measurements each time, with a 1 min interval between them.

**Table 3:** Lifestyle recommendations in accordance with the European Society of Cardiology Guidelines for managing elevated blood pressure and hypertension; DASH: Dietary Approaches to Stop Hypertension.

Lifestyle Recommendation	Class of Recommendation
Reduce dietary sodium intake to less than 2 g/day, equivalent to 5 g or less of salt per day.	IA
Adopt a healthy diet such as the Mediterranean diet or DASH.	IA
Avoid consuming simple sugars, such as sugary drinks. Intake should not exceed 10% of daily energy intake.	IB
Avoid alcohol or limit intake to less than 100 g per week, noting that one drink typically contains 8–14 g of alcohol.	IIaB
Engage in 150 min of moderate-intensity aerobic exercise per week (at least 30 min, 5–7 days a week) or 75 min of vigorous-intensity exercise three days a week, supplemented by moderate to low-intensity dynamic or isometric resistance training 2–3 times a week to reduce hypertension and cardiovascular disease risk.	IA
Smoking Cessation.	IA

## Data Availability

No new data were generated.

## Abbreviations

The following abbreviations are used in this manuscript:

ABPM	Ambulatory blood pressure monitoring
ACE-I	Angiotensin-converting enzyme inhibitors
ADL	Daily activities
ARBs	Angiotensin II receptor blockers
CCB	Calcium channel blockers
CVD	Cardiovascular Disease
DASH	Dietary Approaches to Stop Hypertension
DBP	Diastolic Blood Pressure
ECG	Electrocardiogram
ESC	European Society of Cardiology
ESH	European Society of Hypertension
HBPM	Home blood pressure monitoring
HMOD	Hypertension-mediated organ damage
HT	Arterial Hypertension
MDPI	Multidisciplinary Digital Publishing Institute
MH	Masked Hypertension
MMSE	Mini-Mental State Examination
RH	Resistant Hypertension
SBP	Systolic Blood pressure
WCH	White-coat Hypertension
